# Inhibition of the HER2-YB1-AR axis with Lapatinib synergistically enhances Enzalutamide anti-tumor efficacy in castration resistant prostate cancer

**DOI:** 10.18632/oncotarget.3602

**Published:** 2015-03-15

**Authors:** Masaki Shiota, Jennifer L. Bishop, Ario Takeuchi, Ka Mun Nip, Thomas Cordonnier, Eliana Beraldi, Hidetoshi Kuruma, Martin E. Gleave, Amina Zoubeidi

**Affiliations:** ^1^ The Vancouver Prostate Centre and Department of Urologic Sciences, University of British Columbia, Vancouver, British Columbia, Canada; ^2^ Department of Urologic Sciences, University of British Columbia, Vancouver, British Columbia, Canada

**Keywords:** enzalutamide, HER2, castration resistant prostate cancer, lapatinib

## Abstract

Incurable castration-resistant prostate cancer (CRPC) is driven by androgen receptor (AR) activation. Potent therapies that prevent AR signaling, such as Enzalutamide (ENZ), are mainstay treatments for CRPC; however patients eventually progress with ENZ resistant (ENZR) disease. In this study, we investigated one mechanism of ENZ resistance, and tried to improve therapeutic efficiency of ENZ. We found HER2 expression is increased in ENZR tumors and cell lines, and is induced by ENZ treatment of LNCaP cells. ENZ-induced HER2 overexpression was dependent on AKT-YB1 activation and modulated AR activity. HER2 dependent AR activation in LNCaP and ENZR cells was effectively blocked by treatment with the EGFR/HER2 inhibitor Lapatinib, which reduced cell viability and increased apoptosis. Despite efficacy *in vitro*, *in vivo* monotherapy with Lapatinib did not prevent ENZR tumor growth. However, combination treatment of Lapatinib with ENZ most effectively induced cell death in LNCaP cells *in vitro* and was more effective than ENZ alone in preventing tumor growth in an *in vivo* model of CRPC. These results suggest that while HER2 overexpression and subsequent AR activation is a targetable mechanism of resistance to ENZ, therapy using Lapatinib is only a rational therapeutic approach when used in combination with ENZ in CRPC.

## INTRODUCTION

Prostate cancer (PCa) is the most common male cancer in North America and 2^nd^ leading cause of cancer deaths [1]. While many gains have been made in early detection and treatment of localized PCa, many men still die of recurrent, metastatic disease. This occurs when androgen deprivation therapy (ADT), the standard treatment for advanced localized and metastatic PCa, fails. Androgens are principal factors in PCa carcinogenesis and progression, regulating gene and signaling networks that promote cell survival through binding with the androgen receptor (AR), a ligand-responsive transcription factor. ADT therefore, including medical or chemical castration and the use of non-steroidal anti-androgens, blocks the growth-promoting effects of androgens and activates apoptosis in PCa tumors cells [2], prolonging median overall survival for PCa patients [3]. Despite high initial response rates, remissions following ADT are temporary due to the emergence of castration-resistant prostate cancer (CRPC), where tumors grow in the presence of low levels of androgens.

Because AR activation remains a central mechanism driving CRPC progression [4], targeting the AR with anti-androgen drugs remains a critical component of novel CRPC therapies [5, 6]. Enzalutamide (ENZ) is a second generation anti-androgen that has significant anti-tumor effects both *in vitro* and *in vivo*. Despite the fact that ENZ does improve survival in CRPC patients, it does not lead to complete regression, with tumors recurring in a mean time of 47 weeks [7]. Therefore, despite the efficacy of ENZ in the short term, the development of ENZ resistant (ENZR) tumors in CRPC represents a significant challenge in the treatment of advanced PCa. As such, studying the mechanisms driving resistance to ENZ is critical to improve the future of PCa therapy. In this study, we tried to resolve the importance of the HER2 signaling pathway as mechanism of resistance to ENZ and evaluate the potential of targeting HER2 to improve the therapeutic efficacy of ENZ.

Previous data from our laboratory suggests that as in CRPC, re-activation of the AR occurs during ENZ resistance [8]. In androgen deprived conditions, continued AR activity can result from variable combinations of AR gene amplification, increased AR sensitivity, promiscuous AR binding mutants, altered expression of co-regulators, increases in androgen biosynthesis as well as ligand independent activation by oncogenic signaling pathways [9-12]. In particular, there is increasing evidence suggesting that activation of the EGFR/HER2 pathway is important in driving PCa progression. Multiple reports have indicated that HER2 can activate the AR in androgen deprived conditions [13-15]. Furthermore, EGFR/HER2 expression levels increase with PCa disease progression to CRPC [16] and are also highly upregulated in lymph node metastases [17]. Interestingly however, although targeting of EGFR/HER2 can suppress prostate tumor xenograft growth in *in vivo* models [18, 19], trials using EGFR/HER2 inhibitors such the EGFR inhibitor Gefetinib [20] or the dual EGFR/HER2 inhibitor Lapatinib [21] as single agents in patients with CRPC do not improve overall survival or decrease PSA (a surrogate marker of AR activity). These studies suggest therefore, that HER2 activation of AR signaling is potentially a mechanism of resistance to ENZ and that combination therapy using potent anti-androgens like ENZ with HER2 targeting agents may be a more viable way to prevent AR-reactivation in CRPC patients.

Using ENZR tumor cell lines and LNCaP cells treated with ENZ, we found that HER2 overexpression is both associated with ENZ resistance and a consequence of ENZ treatment. In addition, our data indicates that ENZ-mediated HER2 expression is dependent on the transcription factor YB-1 and that HER2 controls AR activation, potentially through a feed forward mechanism of upregulation of AKT, which is known to activate both YB-1 and the AR itself. Indeed we show that the EGFR/HER2 inhibitor Lapatinib prevented AR activation in both LNCaP and ENZR cell lines and reduced cell viability. While ENZR cell lines were more susceptible *in vitro* to Lapatinib, monotherapy *in vivo* was ineffective in preventing ENZR tumor growth. However, in our *in vivo* model of CRPC, combination therapy of Lapatinib with ENZ was more effective in preventing tumor growth than ENZ treatment alone. Taken together these data provide proof-of-principle that combination therapy using ENZ with Lapatinib may be a viable treatment strategy for CRPC.

## RESULTS

### HER2 overexpression is associated with ENZ treatment and resistance in prostate cancer

**Figure 1:**
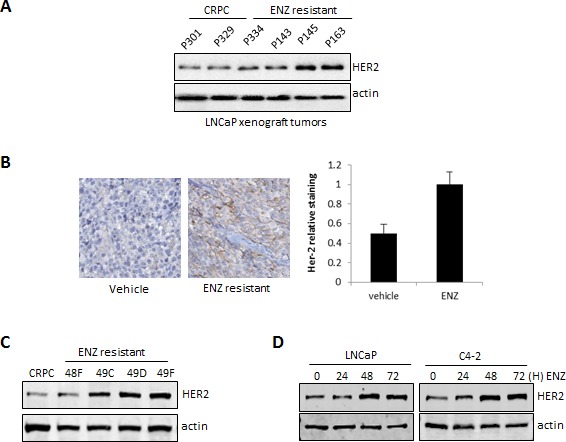
HER2 is overexpressed in ENZ-resistant tumors and cells and induced by ENZ A. Extracts from ENZR and ENZ-sensitive or LNCaP CRPC tumors were analyzed by western blot for expression of total HER2 and β-actin (loading control). B. ENZR and CRPC tumors were stained for HER2 by IHC and expression levels were scored based on levels of immunostaining. For quantification of HER2 staining (right panel) n=9 C. Whole-cell extracts from C4-2 cells and cell lines derived from ENZR and CRPC tumors were analyzed by western blot analysis for the expression of HER2 and β-actin (loading control). D. LNCaP and C4-2 cells were treated with 10 μmol/L of ENZ for 0, 24, 48 and 72 hours and whole-cell extracts were analyzed by western blot analysis for expression of HER2 and β-actin (loading control).

Hyperactivation of oncogenic signaling pathways including HER2 have been implicated as mechanisms driving re-activation of the AR in CRPC and thus contribute to resistance to anti-androgen therapies [16, 17]. We found that HER2 was up-regulated in ENZR tumors compared to CRPC controls tumors (Fig. 1A). Immunohistochemistry analysis also showed that HER2 is highly up-regulated in ENZR tumors compared to CRPC (Fig. 1B). Accordingly, HER2 expression was highly expressed at the protein level in ENZ-resistant cell lines established from ENZ-resistant tumors compared to cell lines derived from CRPC tumors or the prostate cancer cell line C4-2 (Fig. 1C). In addition, we found that ENZ induces HER2 in a time-dependent manner in castrate-sensitive LNCaP and castrate-resistant C4-2 cells (Fig. 1D). Taken together, these results suggest that treatment of PCa with the anti-androgen ENZ increases HER2 expression, which may be a mechanism of therapy resistance.

### ENZ induces HER2 via AKT-YB1 signaling

**Figure 2:**
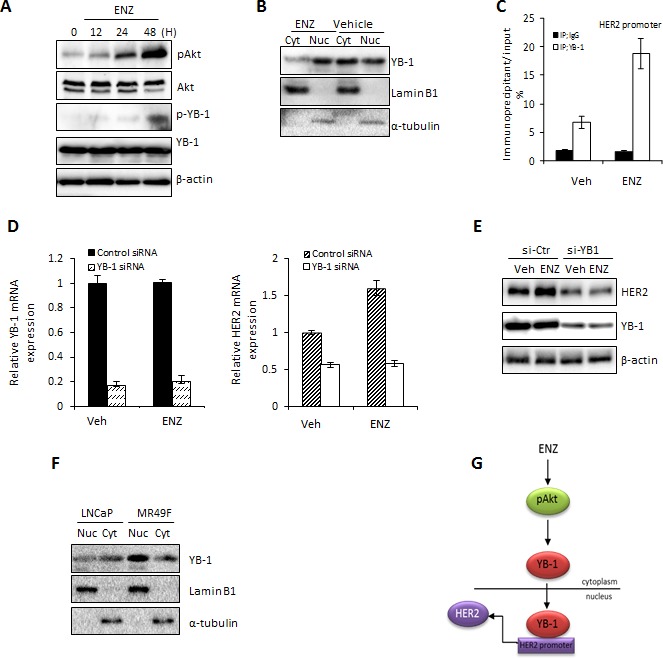
ENZ induces upregulation of HER2 via YB1 A. LNCaP cells were treated with 10 μmol ENZ for 0, 12, 24 or 48 h and expression of phospho- or total YB1 and AKT were assessed by western blot (β-actin loading control). B. LNCaP cells were treated with 10 μM ENZ for 48 h and cell lysate was fractioned into nuclear and cytoplasmic extracts. Expression of YB-1 and laminB1 or α-tubulin (as loading controls) were assessed by western blot. C. ChIP assays were performed on nuclear extracts from LNCaP cells treated with 10 μM ENZ for 48 h using rabbit IgG or anti-YB-1 antibody. qRT-PCR was performed using immunoprecipitated DNAs, soluble chromatin and specific primer pair for the *HER2* promoter region normalized to GAPDH. IgG binding to HER2 in untreated cells are set to 1. Means of a representative experiment is shown ±s.d. D and E. LNCaP cells were transfected with 40 nM of control siRNA or YB-1 siRNA. After 24 h post-transfection, LNCaP cells were treated +/− 10 μM ENZ for 48 h and expression of HER2 and YB-1 at the mRNA and protein level was assessed. For qRT-PCR, target gene expression was normalized to GAPDH, untreated cells were set to 1. Representative experiments (+/− s.d. for RT-PCR) are shown. F. LNCaP and ENZR MR49F cells were treated with 10 μM ENZ for 48 h and cell lysate was fractioned into nuclear and cytoplasmic extracts. Expression of YB-1 and laminB1 or α-tubulin (as loading controls) were assessed by western blot. G. Proposed model showing integration of HER2, YB-1 and ERK1/2 activation as a mechanism of resistance to ENZ.

To investigate the molecular mechanism by which ENZ may upregulate HER2 expression in PCa cells, we assessed the activity of the AKT/YB-1 signal transduction pathway. Previous reports have shown that ENZ induces activation of AKT [22]; in turn, activated AKT leads to phospho-activation of the transcription and translation factor YB-1 [23]. Since YB-1 binds to the promoter of HER2 [24] leading to its increased expression, we hypothesized that ENZ increases HER2 by activating AKT/YB1. Indeed, we found that in LNCaP cells ENZ induced phosphorylation of AKT in a time dependent manner with concomitant increase of YB-1 phosphorylation (Fig. 2A). Accordingly, phosphorylation of YB-1 was associated with ENZ-induced YB-1 nuclear translocation (Fig. 2B), implicating its ability to function as a transcription factor. To investigate whether YB-1 is required for HER2 expression after ENZ treatment, we first assessed YB-1 binding to the HER2 promoter region previously identified as being critical for HER2 transcription by YB-1 [25]. ENZ treatment increased binding of YB-1 to the HER2 promoter as measured by ChIP assay (Fig. 2C), suggesting that YB-1 was required for increased levels of HER2 under these conditions. Further validating our hypothesis that YB-1 activated by ENZ is required for HER2 expression, we found that targeting YB-1 with siRNA abrogated ENZ induced HER2 upregulation at the mRNA (Fig. 2D) and protein level (Fig. 2E). Moreover, we observed predominantly nuclear YB-1 localization in the ENZR cell line MR49F compared to LNCaP (Fig. 2F). Overall, these results suggest that ENZ induces AKT phosphorylation which will activate YB-1, and trigger its nuclear translocation. This allows YB-1 to act as a transcription factor that binds the Y-box in HER2 to activate HER2 expression (Fig. 2G).

### HER2 inhibition by Lapatinib blocks AR activation

**Figure 3:**
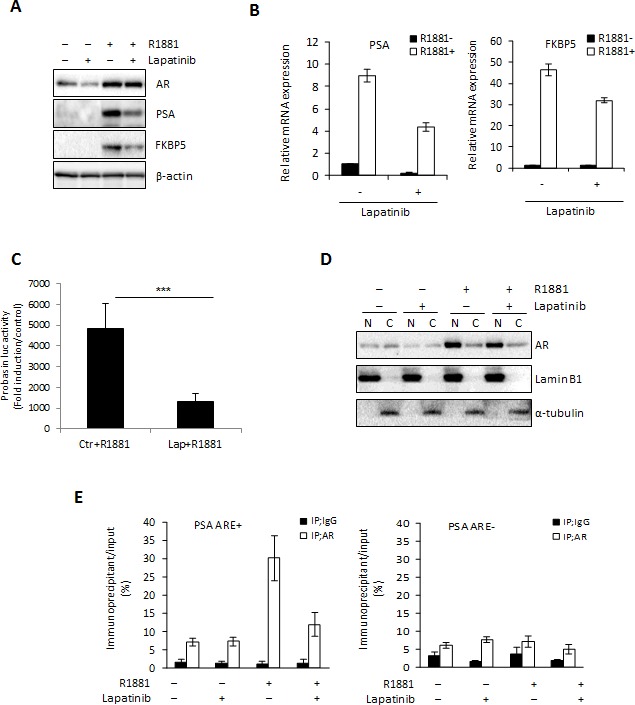
Lapatinib blocks AR activation A-D. LNCaP cells were cultured in serum-free media +/− 10 nM of the synthetic androgen R1881 for 12 h, followed by further incubation +/− 10 μmol Lapatinib for 24 h and AR, PSA and FKBP5 expression in total protein or mRNAwas assessed by western blot (A) or qRT-PCR (B). Means of representative experiments are shown ±s.d. For qRT-PCR target gene expression was normalized to GAPDH and untreated cells set to 1. For western blot analysis β-actin was used as a loading control. C. LNCaP cells were transfected with 0.5 μg/mL of PSA–Luc plasmid and 0.05 μg/mL of pRL-TK. After 12 h, media was changed into serum-free media with or without 10 nmol R1881 for 12 h, followed by further incubation +/− 10 μM Lapatinib or ENZ for 24 h. Fold induction of Probasin-Luc compared to untreated cells is shown. A representative experiment is shown ±s.d. D. LNCaP cells were cultured in serum-free media +/− 10 nmol of the synthetic androgen R1881 for 12 h, followed by further incubation +/− 10 μM Lapatinib for 24 h and cell lysate was fractioned into nuclear and cytoplasmic extracts. Expression of AR and laminB1 or α-tubulin (as loading controls) were assessed by western blot. E. ChIP assay was performed on nuclear extracts from LNCaP cells cultured in serum-free media with or without 10 nmol R1881 for 12 h, followed by further incubation with or without 10 μmol Lapatinib for 1 h using 2.0 μg of rabbit IgG or anti-AR antibody. The quantitative RT-PCR was performed using immunoprecipitated DNAs, soluble chromatin and specific primer pairs for the *PSA* gene normalized to GAPDH. Untreated cells are set to 1. A representative experiment is shown ±s.d.

One important consequence of increased HER2 signaling in CRPC is re-activation of the AR [15, 26, 27], which also occurs during ENZ resistance [8]. Our results showing increased HER2 expression in ENZR cell lines suggest that as in CRPC, HER2 activation of the AR may be a mechanism of resistance to ENZ. Therefore, we examined the effect of Lapatinib, a dual EGFR/HER2 inhibitor, on AR signaling. As expected, we found that Lapatinib treatment induced a decrease of AR regulated genes at the protein and mRNA level in LNCaP cells (Fig. 3A-B) and PSA promoter activity was also significantly reduced in Lapatinib treated LNCaP cells after androgen stimulation (Fig. 3C). This was associated with decreased nuclear AR in Lapatinib treated cells (Fig. 3D). Accordingly, Lapatinib also inhibited androgen mediated AR binding to the androgen-responsive element (ARE) in the promoter region of *PSA* (PSA ARE+) (Fig. 3E). These results indicate that suppression of EGFR/HER2 signaling with Lapatinib inhibits AR activity in PCa cells and suggest the increased dependence of HER2 mediated AR activation in ENZR cells.

### Lapatinib treatment is most effective as a combination therapy with ENZ in CRPC

**Figure 4:**
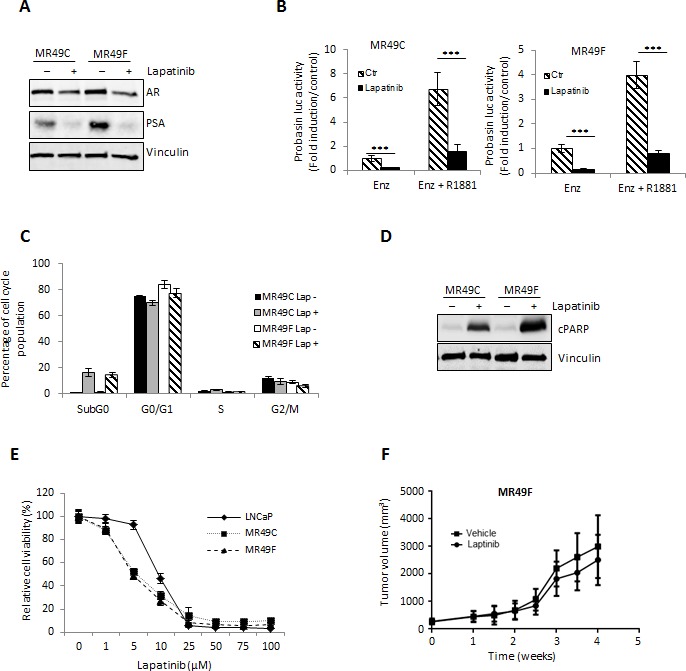
Lapatinib effectively inhibits AR activation and proliferation *in vitro* of ENZR cells A. ENZR MR49C and MR49F cells were cultured in serum-free media with 10uM ENZ +/− 10 μM Lapatinib for 24 h and AR and PSA expression was assessed by western blot. B. LNCaP cells were transfected with 0.5 μg/mL of Probasin–Luc plasmid and 0.05 μg/mL of pRL-TK. After 12 h, media was changed into serum-free media with or without 10 nmol R1881 for 12 h, followed by further incubation +/− 10 μM Lapatinib or ENZ for 24 h. Fold induction of Probasin–Luc compared to untreated cells is shown. A representative experiment is shown ±s.d. C. ENZR MR49F and 49C cells were treated with 10 μM Lapatinib. After incubation for 48 h, the cells were stained with propidium iodide and analyzed by flow cytometry for cell cycle fractions. A representative experiment is shown, error bars ± s.d. D. Whole-cell extracts from LNCaP, MR49F and MR49C cells treated +/− 10 μmol/L Lapatinib for 48 h were analyzed by SDS–PAGE and western blot analysis for cleaved and total caspase 3 and vinculin (loading control). E. LNCaP and ENZR MR49C and MR49F cells were treated with various concentrations Lapatinib for 72 h and cell survival was analyzed by crystal violet assay. Means from a representative experiment are shown, error bars ± s.d. F. After establishment of tumors (200mm^3^) from subcutaneous injection of ENZR MR49F cells in castrated mice under the pressure of 10mg/kg daily of ENZ, mice were treated with 100mg/kg Lapatinib or vehicle alone, n=10. Representative data of tumor volume over 4 weeks is shown.

Our results showing reduction in AR activity by Lapatinib suggested that Lapatinib may improve efficacy of ENZ or target ENZ resistance. To investigate this hypothesis further we assessed PCa cell growth after Lapatinib treatment *in vitro* and *in vivo*. As in LNCaP cells, Lapatinib treated ENZR MR49C and MR49F cells showed a reduction in AR and PSA expression (Fig. 4A). Probasin luciferase activity in both ENZR cell lines was also significantly inhibited by Lapatinib, in both androgen stimulated and unstimulated conditions (Fig. 4B). Reduced AR signaling in ENZR cells treated with Lapatinib was associated with increased frequency of cells in the SubG0 phase of cell cycle (Fig. 4C) and Lapatinib treatment induced PARP cleavage (Fig. 4D). Accordingly, Lapatinib effectively reduced viability of ENZR MR49C and MR49F cells *in vitro* and these cells were more sensitive than LNCaP cells to treatment (Fig. 4E).

Our *in vitro* results further support the hypothesis that HER2 mediated AR activation is a mechanism of resistance to ENZ and suggest that this mechanism can be overcome by targeted HER2 inhibition with Lapatinib. To investigate this possibility further, we questioned whether Lapatinib would be an effective monotherapy against ENZR tumors *in vivo*. Contrasting our *in vitro* results, we found that Lapatinib had no effect on growth of ENZ resistant MR49F tumors in castrated male mice compared to vehicle alone (Fig. 4F). While in contrast to our *in vitro* findings, these results support previous data from our laboratory which showed that further targeting the AR in ENZ resistant MR49F tumors *in vivo* provides only short-lived benefit in terms of slowing tumor growth [8] as well as data from clinical trials showing that monotherapy with Lapatinib is not effective in PCa patients [21, 33]. Taken together, these data suggest that while single agent targeting of mechanisms of ENZ resistance may reduce cell viability *in vitro*, they are not sufficient to prevent tumor progression *in vivo*.

**Figure 5:**
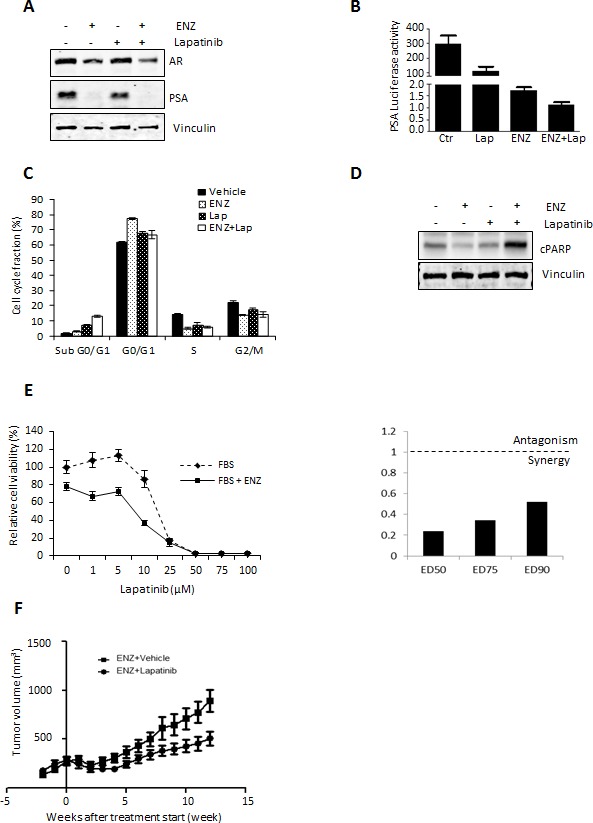
Lapatinib augments therapeutic effect of ENZ *in vitro* and *in vivo* A. LNCaP cells were cultured in serum-free media +/− 10 nM of the synthetic androgen R1881 for 12 h, followed by further incubation +/− 10 μM Lapatinib and/or 10uM ENZ for 24 h and AR and PSA was assessed by western blot. B. LNCaP cells were transfected with 0.5 μg/mL of PSA–Luc plasmid and 0.05 μg/mL of pRL-TK. After 12 h, media was changed into serum-free media with or without 10 nmol R1881 for 12 h, followed by further incubation +/− 10 μM Lapatinib and/or ENZ for 24 h. Fold induction of Probasin–Luc compared to untreated cells is shown. A representative experiment is shown ±s.d. C. LNCaP cells were treated with 10 μM of ENZ and/or Lapatinib. After incubation for 48 h, the cells were stained with propidium iodide and analyzed by flow cytometry for cell cycle fractions. Means from a representative experiment are shown, error bars ± s.d. D. Whole-cell extracts from LNCaP cells treated with 20 μmol/L of ENZ and/or Lapatinib for 48 h were analyzed by western blot analysis for cleaved PARP and β-actin (loading control). E. (Right) LNCaP cells were treated with various concentrations Lapatinib for 72 h and cell survival was analyzed by crystal violet assay. Means from a representative experiment are shown, error bars ± s.d. (Left) Dose-dependent effects of Lapatinib, ENZ or Lapatinib+ENZ (right panel) and CI values for ED50, ED75 and ED90 of Lapatinib+ENZ (left panel) calculated by CalcuSyn software were assessed. Cell growth in the absence of inhibitor corresponds to 1. F. After establishment of LNCaP tumors (200mm^3^) in castrated mice, mice were treated with 100mg/kg Lapatinib or vehicle alone, n=10. Representative data of tumor volume over 12 weeks is shown.

As an alternative to monotherapy, combination therapies targeting novel signaling pathways along-side the AR may be more viable alternatives for patients with anti-androgen resistant PCa [28]. To investigate this possibility, we assessed the therapeutic combination effect of ENZ with Lapatinib on AR signaling in LNCaP cells. As shown in Fig. 5A, combination treatment of Lapatinib with ENZ enhanced the reduction of AR and PSA expression (Fig. 4A) as well as AR transactivation in LNCaP cells (Fig. 4B) more so than ENZ or Lapatinib treatment alone. Accordingly, combination of ENZ with Lapatinib also showed the greatest efficacy in increasing the fraction of cells in the SubG0 phase of cell cycle (Fig. 4C) and induction of PARP cleavage (Fig. 4D) compared to ENZ or Lapatinib alone. Moreover, combination treatment of ENZ and Lapatinib showed the greatest reduction in cell viability compared to monotherapy with Lapatinib. Calculated combination indices determined at ED_50_, ED_75_ and ED_90_ revealed values below 1 (Fig. 5E), indicating ENZ and Lapatinib are strongly synergistic in LNCaP cells. These results suggest that HER2 signaling is important for growth and survival in the context of anti-androgen treatment and co-targeting this pathway and the AR may have synergistic effects in CRPC.

To explore whether Lapatinib would improve efficacy of ENZ in CRPC, LNCaP tumors were xenografted subcutaneously into male nude/scid mice and after castration resistant recurrence of LNCaP tumors, mice were treated with ENZ alone, or combination therapy of ENZ with Lapatinib. Supporting our hypothesis that combination treatment targeting HER2 with anti-androgen may be an effective treatment for CRPC, our results showed that adding Lapatinib treatment to ENZ significantly reduced tumor volume compared to animals treated with ENZ alone (Fig. 5F). Overall our *in vitro* and *in vivo* results suggest that ENZ induced upregulation of HER2 occurs via AKT/YB-1 signaling, leads to AR activation and can effectively be targeted to improve therapeutic efficacy of ENZ.

## DISCUSSION

Overcoming treatment resistance in CRPC is essential to improve therapy for patients who no longer respond to even the most effective anti-androgen treatment. Although inhibition of AR signaling will always be a mainstay of PCa treatments, there is an unmet clinical need to use therapies that target signaling pathways that are induced by hormonal therapy and/or contribute to AR re-activation in resistant disease. In this study, we show that treatment with ENZ, the most recently approved anti-androgen, increases activity of the HER2 signaling pathway and hyperactivity of HER2 is a mechanism of resistance to ENZ that can be overcome using targeted HER2 inhibition by Lapatinib. Moreover, we provide a mechanism for not only ENZ induced HER2 upregulation, but also HER2 control of AR signaling via the transcription factor YB-1. These results support many clinical findings that draw correlations between HER2 and YB-1 expression with progression of PCa to CRPC and provide proof-of-principle for combination therapy using Lapatinib with ENZ in the future.

EGF signaling has been implicated in PCa progression in a variety of reports. Activation of EGF receptor and HER2 signaling is a known consequence of treatment with previous generation anti-androgens in human and mouse [29, 30] and increased expression of EGFR or HER2 correlated with disease relapse, progression to CRPC and poor prognosis [16, 31]. We observed that LNCaP cells treated with the potent anti-androgen ENZ support these previous reports, with these cells showing increased expression of HER2. Moreover, our data in newly developed ENZR xenografts and cell lines showing overexpression of HER2 suggested that HER2 activity may contribute to treatment resistance, providing support for investigating the efficacy of HER2 inhibitors after ENZ treatment and in ENZR cells. Indeed, we found that combining Lapatinib, a dual EGFR/HER2 inhibitor, with ENZ most effectively reduced cell viability in LNCaP cells *in vitro* and suppressed tumor growth vivo, supporting previous findings showing HER2 inhibition augments the therapeutic effect of androgen deprivation in PCa cells [32].

AR activity is the primary source of proliferation and survival signals in PCa tumor cells, both in primary disease and in CRPC. Thus, we sought to determine whether hyperactivity of HER2 in response to ENZ was required for AR signaling. In fact, it is well known that EGFR/HER2 signaling can constitutively activate AR and render prostate cancer cells refractory to AR blockade [15, 26]. Our results showing that ENZR cells, which had the highest levels of HER2 expression, were even more susceptible to Lapatinib treatment than LNCaP cells support these results and suggest that ENZR cells are dependent on HER2 mediated activation of the AR for survival. Indeed, treatment with Lapatinib abrogated the androgen-induced transcription and translation of downstream AR target genes and ENZR cells were more susceptible to Lapatinib treatment *in vitro* than LNCaP. Despite these strong effects observed on ENZR cells *in vitro* however, targeting growth of ENZR tumors *in vivo* with Lapatinib monotherapy was not sufficient to prevent tumor progression. This is an important finding, which indicates that although HER2 may indeed drive further AR activation as a mechanism of ENZ resistance, targeting this single pathway is not effective against late stage disease and echo previous reports showing that monotherapy with HER2 inhibitors have been unsuccessful in PCa [21, 33]. By contrast however, Lapatinib combination therapy with ENZ in our model of CRPC was effective in slowing tumor growth. This effect may be due to the ability of combination treatment to work most efficiently in reducing AR activity in LNCaP cells. Indeed, in androgen stimulated LNCaP cells, combination treatment of Lapatinib and ENZ reduced AR transactivation activity by approximately 270 fold, whereas transactivation in ENZR cells was only reduced by 5 fold. These results suggest that in response to androgen deprivation, PCa cells can activate the AR to promote survival and proliferation via HER2 and that targeting this mechanism of survival can increase efficacy of ENZ in CRPC, but not overcome treatment resistance.

Investigation into the mechanism by which HER2 is activated by ENZ indicated the importance of the AKT/YB-1 signaling pathway. The Y-Box binding protein 1 (YB-1) is a transcription factor that positively correlates with HER2 expression in breast cancer [34] and lung cancer [35]. Previous reports have shown that HER2 expression is regulated by YB-1 in breast cancer cells [25] and that YB-1 up-regulates HER2 expression by HER2 gene amplification [36]. Our results support the requirement for YB-1 in mediating HER2 expression in PCa as well, especially in androgen deprived conditions, as we found that YB-1 bound to the HER2 promoter in LNCaP cells and YB-1 was required for ENZ mediated upregulation of HER2. The activation of YB-1 in our system was induced by ENZ downstream of AKT, supporting previously published results [37].

The correlation between increased YB-1 and HER2 expression after ENZ treatment not only provides a mechanism for HER2 upregulation itself, but also a possible mechanism of AR activity regulation by HER2. Like re-activation of the AR, YB-1 expression is closely associated with aggressive PCa. Total and nuclear YB-1 expression in human PCa increases with Gleason Grade and upon treatment with androgen deprivation therapy [38, 39]. In addition, our previous results indicate that YB-1 is required for AR activation in models of CRPC [40]. These studies in PCa support findings in other cancers where YB-1 mediates treatment resistance [38, 41]. Based on these data and our own presented herein, we hypothesize that ENZ treatment initiates a feed forward loop involving YB-1, HER2, and AR activation that contributes to treatment resistance.

In conclusion, we have identified overexpression of HER2 as a mechanism of resistance to the potent anti-androgen ENZ. Our results in ENZR xenografts and cell lines as well as LNCaP cells treated with ENZ indicated that YB-1 and AKT are also upregulated in response to treatment, which may contribute to HER2 mediated activation of the AR, promoting cell survival. YB-1 was also identified as being required for HER2 upregulation, implicating a convergence of oncogenic signaling pathways upon the AR that can mediate treatment resistance. Most importantly, our studies *in vitro* and *in vivo* provide proof-of-principle for the rational combination of HER2 targeted therapy, such as Lapatinib, with ENZ to treat CRPC and delay the emergence of ENZR disease.

## MATERIALS AND METHODS

### Cell culture and transfection

The human prostate cancer LNCaP cells were kindly provided by Dr. Leland W.K. Chung (1992, MD Anderson Cancer Center, Houston, TX), tested and authenticated by whole-genome and whole-transcriptome sequencing on Illumina Genome Analyzer IIx platform in 2013. LNCaP cells were maintained in RPMI 1640 (Thermo Scientific, Burlington, ON) supplemented with 5% FBS. ENZ-resistant cells (MR49C and MR49F) derived from LNCaP cells were established and maintained as described previously [8, 28, 42].

### Antibodies and reagents

Antibodies against AR (sc-816), prostate-specific antigen (PSA; sc-7638) and FKBP5 (sc-11514) were purchased from Santa Cruz Biotechnology (Santa Cruz, CA). anti-HER2 (#2165), anti-phosphorylated Y-box binding protein-1^Ser102^ (p-YB-1; #2900), anti-α-tubulin (#2125), anti-Akt (#9272), anti-phosphorylated Akt^Ser473^ (p-Akt; #4060), anti-PARP (#9542), and anti-cleaved PARP (#9541) antibodies were obtained from Cell Signaling (Danvers, MA). Antibodies against Lamin B1 (ab16048) were purchased from Abcam (Cambridge, MA). Anti-YB-1 (2397-1) antibodies were obtained from Epitomics (Burlingame, CA). Anti-β-actin (A3854) antibodies were purchased from BD Biosciences (Franklin Lakes, NJ) and Sigma (St Louis, MO).

### siRNAs

The following double-stranded 25-bp siRNA oligonucleotides were commercially generated (Invitrogen, Carlsbad, CA): 5′-UUUGCUGGUAAUUGCGUGGAGGACC-3′ for YB-1 siRNA. Stealth™ RNAi Negative Control Medium GC Duplex #2 (Invitrogen Life Technologies, Inc., Carlsbad, CA) was used as a control siRNA.

### Quantitative reverse transcription (RT)-PCR

RNA extraction and RT-PCR were performed as previously described [28]. Real time monitoring of PCR amplification of cDNA was performed using the following primer pairs and probes: *AR* (Hs00171172_m1), *PSA* (Hs00426859_g1), *FKBP5* (Hs01561006_m1), *YB-1* (Hs00898625_g1) and *GAPDH* (Hs03929097_g1) (Applied Biosystems, Foster City, CA) on ABI PRISM 7900 HT Sequence Detection System (Applied Biosystems) with TaqMan Gene Expression Master Mix (Applied Biosystems). Target gene expression was normalized to *GAPDH* levels in respective samples as an internal control.

### Western blot analysis

Whole-cell extracts were obtained by lysis of cells in an appropriate volume of ice-cold RIPA buffer composed of 50 mmol/L Tris-HCl (pH 7.4), 150 mmol/L NaCl, 0.5% sodium deoxycholate, 1% Nonidet P-40, 0.1% sodium dodecyl sulfate (SDS) containing 1 mmol/L Na_3_VO_4_, 1 mmol/L NaF, 1 mmol/L phenylmethylsulfonyl fluoride and protease inhibitor cocktail tablets (Complete, Roche Applied Science, Indianapolis, IN). Nuclear and cytoplasmic extracts were obtained using CelLytic^TM^ NuCLEAR^TM^ Extraction Kit (Sigma) according to manufacturer's protocol. Extracts were clarified by centrifugation at 13,000 x *g* for 10 min and protein concentrations of the extracts determined by a BCA protein assay kit (Thermo Scientific). Extracts (30 μg) were boiled for 5 min in SDS sample buffer and separated by SDS-PAGE, and transferred onto a polyvinylidene difluoride (PVDF) membrane. Membranes were probed with diluted primary antibodies followed by incubation with horseradish peroxidase-conjugated secondary antibodies. After extensive washing, proteins were visualized by a chemiluminescent detection system (GE Healthcare, Buckinghamshire, UK).

### Immunohistochemistry

Immunohistochemical staining was conducted using the Ventana Discover XT Autostainer (Ventana Medical System) on paraffin embedded tumors with enzyme labeled biotin streptavidin system and a solvent-resistant DAB Map kit by using HER2 antibody. Slides were scored as follows: 0= no staining, 1= faint or focal stain, 2= convincing intensity in a minority of cells, and 3= convincing intensity in a majority of cells. The overall percentage of cancer cells showing staining (0%–100%) was also indicated. Scoring was conducted at 200x.

### Luciferase reporter assay

Luciferase reporter assay was performed as described previously [8]. Briefly, LNCaP cells were transfected with 1.0 μg/mL of PSA reporter plasmid (PSA-Luc) or Probasin reporter plasmid and 0.05 μg/mL of pRL-TK as an internal control. After 12 h from transfection, media was changed to serum-free media with or without 10 nmol/L of R1881. At 12 h later, cells were further incubated with or without 10 μmol/L of Lapatinib for 24 h. The luciferase activities were measured using a Dual-Luciferase Reporter Assay System (Promega, Madison, WI) and a microplate luminometer (EG&G Berthold, Bad Wilbad, GER). The *Firefly* luciferase activities were c49C, corrected by the corresponding *Renilla* luciferase activities.

### Chromatin immunoprecipitation assay (ChIP assay)

ChIP assay was performed as previously described [9]. Briefly, LNCaP cells were cross-linked with paraformaldehyde and digested with micrococcal nuclease to achieve a DNA smear of 200-1000 bp. ChIP assay was performed using SimpleChIP^TM^ Enzymatic Chromatin IP Kit (Cell Signaling Technology) according to the manufacturer's protocol on the *HER2* or *PSA* gene. The quantitative RT-PCR assay with DNA extraction, the primer pairs below and FastStart Universal SYBR Master (Roche, Missassauga, ON) was performed using ABI 7900HT System (Applied Biosystems). The results are representative of at least three independent experiments. The sequences of primer pairs are as follows: 5′-AGGGGCTCCAAATAGAATGT-3′ (Fw) and 5′-AATTTGGGAGGAGACAGTCA-3′ (Rv) for *HER2* promoter targeting between –978 bp and –514 bp from transcription start site (TSS) [43]; 5′-TCTGCCTTTGTCCCCTAGAT-3′ (Fw) and 5′-AACCTTCATTCCCCAGGACT-3′ (Rv) for PSA ARE+ targeting between –250 bp and –39 bp from TSS; and 5′-CTGTGCTTGGAGTTTACCTGA-3′ (Fw) and 5′-GCAGAGGTTGCAGTGAGCC-3′ (Rv) for PSA ARE– targeting between –1997 bp and –1846 bp from TSS.

### Cell growth assay

LNCaP, MR49C and MR49F cells were plated in 96-well plates and treated with ENZ and/or Lapatinib at indicated concentration. After incubation for 72 h, cell growth was measured using the crystal violet assay as described previously [44]. The combination index (CI) was evaluated using CalcuSyn dose–effect analysis software (Biosoft, Cambridge, UK). This method, based on the multiple drug effect equation of Chou–Talalay [45], is suitable for calculating combined drug activity over a wide range of growth inhibition: CI ¼ 1, additivity; CI > 1, antagonism; CI < 1, synergism. CI was calculated at ED50 and ED75.

### Flow cytometry analysis

LNCaP and MR49F and MR49C cells were plated in 10-cm dishes and treated with ENZ and/or Lapatinib at indicated concentration. After incubation for 48 h, cell-cycle fraction was analyzed as described previously [28].

### Animal studies

Animal treatment was performed as described previously [28, 44]. For CRPC studies, male athymic mice (Sprague Dawley; Harlan, Inc., Indianapolis, IN) were injected subcutaneously with 1 × 10^6^ LNCaP cells (suspended in 0.1 mL Matrigel; BD Biosciences) and castrated when serum PSA increased above 70 ng/mL. For ENZR studies, male athymic mice (Sprague Dawley; Harlan, Inc., Indianapolis, IN) were injected subcutaneously with 2 × 10^6^ ENZR MR49F cells (suspended in 0.1 mL Matrigel; BD Biosciences) under the pressure of 10mg/kg ENZ (daily oral dose). In the CRPC study, once tumors progressed to castrate resistance, mice were randomly assigned to (i) vehicle, (ii) ENZ (10 mg/kg) alone, (iii) Lapatinib (100 mg/kg) alone or (iv) ENZ (10 mg/kg) combined with Lapatinib (100 mg/kg) and treated orally 5 times per week. In the ENZR study, once tumors reached 200mm3, mice were randomly assigned to treatment with (i) vehicle or (ii) 100mg/kg Lapatinib. Tumor volume measurements were performed weekly or twice weekly for CRPC and ENZR studies, respectively. Tumor volume was calculated by the formula: length x width x depth. At experimental endpoint, tumors were harvested and portions were snap frozen for protein analysis or fixed in 10% Neutral Buffered Formalin for IHC analysis. All animal procedures were performed according to the guidelines of the Canadian Council on Animal Care and appropriate institutional certification.
